# Treatment With CD52 Antibody Protects Neurons in Experimental Autoimmune Encephalomyelitis Mice During the Recovering Phase

**DOI:** 10.3389/fimmu.2021.792465

**Published:** 2021-12-16

**Authors:** Wenlin Hao, Qinghua Luo, Michael D. Menger, Klaus Fassbender, Yang Liu

**Affiliations:** ^1^ Department of Neurology, Saarland University, Homburg, Germany; ^2^ Department of Neurology, Diakonie Klinikum Neunkirchen, Neunkirchen, Germany; ^3^ Department of Experimental Surgery, Saarland University, Homburg, Germany

**Keywords:** multiple sclerosis, experimental autoimmune encephalomyelitis, CD52, neuroinflammation, neuroprotection

## Abstract

Multiple sclerosis (MS) is a chronic autoimmune disease driven by T and B lymphocytes. The remyelination failure and neurodegeneration results in permanent clinical disability in MS patients. A desirable therapy should not only modulate the immune system, but also promote neuroprotection and remyelination. To investigate the neuroprotective effect of CD52 antibody in MS, both C57BL/6J and SJL mice with experimental autoimmune encephalomyelitis (EAE) were treated with CD52 antibody at the peak of disease. Treatment with CD52 antibody depleted T but not B lymphocytes in the blood, reduced the infiltration of T lymphocytes and microglia/macrophages in the spinal cord. Anti-CD52 therapy attenuated EAE scores during the recovery phase. It protected neurons immediately after treatment (within 4 days) as shown by reducing the accumulation of amyloid precursor proteins. It potentially promoted remyelination as it increased the number of olig2/CC-1-positive mature oligodendrocytes and prevented myelin loss in the following days (e.g., 14 days post treatment). In further experiments, EAE mice with a conditional knockout of BDNF in neurons were administered with CD52 antibodies. Neuronal deficiency of BDNF attenuated the effect of anti-CD52 treatment on reducing EAE scores and inflammatory infiltration but did not affect anti-CD52 treatment-induced improvement of myelin coverage in the spinal cord. In summary, anti-CD52 therapy depletes CD4-positive T lymphocytes, prevents myelin loss and protects neurons in EAE mice. Neuronal BDNF regulates neuroprotective and anti-inflammatory effect of CD52 antibody in EAE mice.

## Introduction

Multiple sclerosis (MS) is a T and B lymphocytes-mediated chronic autoimmune disease characterized by disseminated demyelination, inflammatory infiltration, and axonal loss in the central nervous system (CNS) (1, 2). In the early phase, axons are relatively preserved, and clinical symptoms often resolve fully due to the neuronal plasticity and partial or complete remyelination. However, the tissue repairing after the disease progress is not sufficient and the remyelination failure is accumulated. Neurons without support of myelin sheath continuously degenerate, which in the end causes permanent clinical disability (1, 3). The desired therapeutic strategies should not only modulate the activity of T and B lymphocytes, but also promote neuroprotection and remyelination in MS patients.

The exact mechanisms mediating axonal loss in MS are incompletely understood; however, it was observed that inflammatory activation compromises mitochondrial function, which impairs energy supply and calcium homeostasis in neurons (4, 5). In chronically demyelinated axons, the loss of myelin support potentially leads to cytoskeletal disorganization and axonal transport deficits (6). T lymphocytes and microglia/macrophages regulate remyelination. Interferon-γ (IFN-γ), produced by T helper 1 (Th1) cells, impairs the differentiation of oligodendrocyte progenitor cells (OPCs) in culture (7). T helper 17 (Th17) cells suppress (8), whereas, interleukin-10 (IL-10)-producing T regulatory cells (Treg) enhance OPC differentiation and remyelination in the brain (9). B lymphocytes participate in MS pathogenesis, producing antibodies, presenting antigens to T cells, and secreting proinflammatory cytokines. However, B cells also function as B regulatory (Breg) cells producing anti-inflammatory cytokines, e.g. IL-10 (2). Intravenous administration of Breg cells expands Treg population, polarizes microglia/macrophages to alternative activation and promotes remyelination in the spinal cord of experimental autoimmune encephalomyelitis (EAE) mice, a popularly used MS animal model (10, 11).

Alemtuzumab is a humanized monoclonal antibody against CD52. By using this antibody circulating T and B lymphocytes including encephalitogenic cells are deleted, and relatively healthy T and B cell populations are thereafter reconstructed in MS patients (12). In phase III clinical trials (CARE-MS I and II trials), treatment with alemtuzumab, especially at a high dose of 24 mg/day, reduces the relapse of relapsing-remitting MS significantly better than the treatment with IFN-β (13–15). Interestingly, alemtuzumab treatments have significantly stronger effects than the treatments with IFN-β to stabilize or improve disability during the MS progression and even after 7-year follow-up (14, 16, 17). Thus, treatments with alemtuzumab might not only prevent relapse of MS, but also establish a neurotrophic microenvironment in MS patients. However, the exact mechanism of action has not been fully elucidated.

In last several years, several studies showed that treatment with CD52 antibodies in myelin oligodendrocyte glycoprotein (MOG)35-55-immunized EAE mice at the early symptomatic stage attenuated the developing symptoms and demyelination (18, 19). However, remyelination was not addressed. In a B cell-dependent EAE mice (MP4-induced EAE model), which were immunized with a fusion protein of myelin basic protein (MBP) and proteolipid protein (PLP), anti-CD52 treatment at the peak disease reserved axons and decreased clinical scores; however, neither demyelination nor remyelination was altered (20). Moreover, CD52 antibody did not directly affect the function of neurons and microglia (21). Thus, further studies are needed to clarify the therapeutic mechanisms of CD52 antibodies.

The T lymphocytes reconstructed in MS patients after treatment of alemtuzumab secret more brain-derived neurotrophic factor (BDNF) (22). BDNF produced from astrocytes and peripheral immune cells have been reported to protect neurons and attenuate EAE symptoms (23, 24). Thus, we would ask whether BDNF mediates the therapeutic effects of CD52 antibodies in MS.

In this project, we treated EAE models established on C57BL/6 and SJL mice with CD52 antibodies at the peak of disease and analyzed MS-associated pathologies, i.e., neuroinflammation, remyelination and neuroprotection, within 4 days and 14 days after treatments. We observed that the deletion of T but not B lymphocytes by administration of CD52 antibodies protects neurons immediately and potentially promotes remyelination at a later stage of disease. BDNF regulates neuroprotection and inflammatory activation in CD52 antibody-treated EAE mice.

## Materials And Methods

### Experimental Animals

C57BL/6J and SJL mice for EAE establishment were originally ordered from Charles River Laboratories (Sulzfeld, Germany). *bdnf* ^fl/fl^ mice carrying loxP-site-flanked exon IX, the single protein coding exon of *bdnf* gene, were kindly provided by M. Sendtner (University of Würzburg) (25). Camk2a-CreERT2 transgenic mice, expressing a fusion protein of Cre recombinase and an estrogen receptor ligand binding domain (CreERT2) under the control of the mouse *calcium/calmodulin-dependent protein kinase II α* promoter, were obtained from the Jackson Laboratory (Bar Harbor, USA; Stock Number: 012362) (26). GFAP-CreERT2 transgenic mice, expressing CreERT2 under the control of human *GFAP* promoter, were kindly provided by F. Kirchhoff, Saarland University (27). Cx3Cr1-CreERT2 knock-in mice, expressing CreERT2 under the control of endogenous *cx3cr1* promoter/enhancer elements (28) were kindly provided by M. Prinz, University of Freiburg. CD4-Cre transgenic mice, expressing Cre under the control of *cd4* gene enhancer/promoter/silencer (29), were kindly provided by A. Waisman, Johannes Gutenberg-University Mainz. To conditionally knock out BDNF in various mice, *bdnf* ^fl/fl^ mice were cross-bred with different Cre recombinase-expressing mice to get *bdnf^fl/fl^/camk2α (GFAP, cx3cr1 or cd4)-cre* ^+/- (or tg)^ and *cre* ^-/- (or wild-type)^ of genotypes. Mice expressing CreERT2 and their compared control littermates without Cre expression were both injected (*i.p.*) at 1 month old with tamoxifen (Sigma-Aldrich Chemie GmbH, Munich, Germany; 100 mg/kg) in corn oil once a day over 5 days. All animal experiments were approved by the regional ethical committee in Saarland, Germany.

To verify the knockout of *bdnf* gene in specific cell lineages, BDNF was co-stained with protein markers of neurons and astrocytes in the spinal cord with immunological methods (see the following method description). Brain tissues of *bdnf^fl/fl^/camk2α-cre* ^tg^ and *bdnf^fl/fl^/camk2α-cre* ^wt^ mice were further homogenized in RIPA buffer (50 mM Tris [pH 8.0], 150 mM NaCl, 0.1% SDS, 0.5% sodiumdeoxy-cholate, 1% NP-40, and 5 mM EDTA) supplemented with protease inhibitor cocktail (Sigma Aldrich Chemie GmbH). The protein level of BDNF was evaluated with quantitative Western blot using rabbit antibodies against BDNF (catalog numbers: NBP1-46750; Novus Biologicals, Wiesbaden-Nordenstadt, Germany) and β-actin (clone 13E5; Cell Signaling Technology Europe, Frankfurt am Main, Germany).

To detect BDNF in CD4-positive T lymphocytes in *bdnf^fl/fl^/cd4-cre* ^tg^ and *bdnf^fl/fl^/cd4-cre^wt^* mice, single cell suspensions were prepared from the spleen. After blocking cells with CD16/CD32 antibodies, T cells were positively selected with Dynabeads® magnetic beads-conjugated rat anti-mouse CD4 antibody (clone: L3T4; Thermo Fisher Scientific, Darmstadt, Germany). As a control, CD11b-positive cells were also selected with rat anti-CD11b monoclonal antibody (clone M1/70; R&D Systems GmbH, Wiesbaden-Nordenstadt, Germany) and Dynabeads® magnetic beads-conjugated sheep anti-rat IgG (Thermo Fisher Scientific). Selected cells were immediately lysed in the lysis buffer from RNeasy Plus Mini Kit (Qiagen, Hilden, Germany).

### Establishment of EAE

Active EAE models were created with our established protocols (30, 31). Eight-week-old C57BL/6J mice, and BDNF-ablated and wild-type littermate mice were *s.c.* immunized with 100μg mouse MOG35-55 (MEVGWYRSPFSRVVHLYRNGK, EZBiolab, Carmel, USA) in a complete Freund’s adjuvant (BD Biosciences, Heidelberg, Germany) supplemented with 100μg Mycobacterium tuberculosis (H37Ra) (BD Biosciences), followed by *i.v.* injection of Pertussis toxin (PTX, Enzo Life Sciences GmbH, Lörrach, Germany) 0 and 2 days after MOG immunization (d.p.i). Eight-week-old SJL mice were immunized using the same protocol as for C57BL/6J mice, except that PLP139-151 (HCLGKWLGHPDKF, EZBiolab) was used as the immunogen.

Assessment of clinical disease activity was performed with the following scores: 0, no disease; 0.5, weak tail or mild hind limb ataxia; 1, limp tail and/or hind limb ataxia; 2, hind limb paresis; 3, hind limb paralysis; 4, hind and fore limb paralysis; 5, death.

### Treatment With CD52 Antibody

BDNF-ablated and wild-type EAE littermate mice with the similar clinical score at the peak of disease, and the same gender and genotype were randomly separated into two groups, which were subcutaneously injected either with anti-mouse CD52 antibody (10 mg/kg body weight; kindly provided by Sanofi Genzyme, Cambridge, MA, USA), or with phosphate-buffered saline (PBS) vehicle for five consecutive days.

### Flow Cytometry Analysis of T and B Cell Populations

Peripheral blood was collected from inferior vena cava and mixed with EDTA. The whole blood (100 µl) was stained with FITC and PE-Cy7-conjugated rat monoclonal antibodies against mouse CD4 (clone GK1.5) and CD19 (clone eBio1D3), respectively (both antibodies were bought from Thermo Fisher Scientific), and then incubated with BD FACS™ lysing solution (BD Bioscience) for the removal of contaminating red blood cells. Immune cell populations were analyzed using flow cytometry (BD FACSCanto II), and absolute numbers of cells were counted using BD Trucount absolute counting tubes (BD Biosciences).

### Tissue Collection

Animals were euthanized at the end of experiments by inhalation of isoflurane. Mice were perfused with ice-cold PBS and the spinal cord was removed. The cervical segment was homogenized in TRIzol (Thermo Fisher Scientific) for RNA isolation and the remainder of spinal cord were immediately fixed in 4% paraformaldehyde (Sigma-Aldrich Chemie GmbH) in PBS and embedded in paraffin for immunohistochemistry.

### Histological Examination

Serial 5µm-thick transverse sections were cut from paraffin-embedded spinal cords and mounted on glass slides. Immunohistochemical staining was performed with the VECTASTAIN *Elite* ABC kit (Vector Laboratories, Burlingame, CA, USA) and primary antibodies against amyloid precursor protein (rabbit anti-APP C-Terminal; catalog number: A8717, Sigma-Aldrich Chemie GmbH), macrophage/microglia (rabbit anti-Iba1; Wako Chemicals, Neuss, Germany), T cells (rabbit polyclonal anti-CD3; catalog number: ab5690, Abcam, Cambridge, UK) and B cells (rabbit monoclonal antibody against CD19; clone D4V4B; Cell Signaling Technology Europe). Sections were developed with diaminobenzidine tetrahydrochloride hydrate (Sigma-Aldrich Chemie GmbH). Moreover, T cells were also stained with CD3 antibody (Abcam) and Cy3-conjugated goat anti-rabbit IgG (Thermo Fisher Scientific). Per animal, four equidistant sections (with a 100 µm interval) of the lumber segment of spinal cord were examined on a Zeiss AxioImager.Z2 microscope (Carl Zeiss Microscopy GmbH, Göttingen, Germany) equipped with a Stereo Investigator system (MBF Bioscience, Williston, ND, USA). APP-positive spheroids, and Iba1 or CD3-positive cells were counted in the white matter of anterior and lateral columns and normalized to the analyzed area (mm^2^).

To quantify cells that are detected by both antibodies against oligodendrocyte transcription factor 2 (olig2) and adenomatous polyposis coli (APC), immunofluorescent staining was performed using rabbit polyclonal antibody against olig2 (catalog numbers: NBP1-28667, Novus Biologicals) and mouse monoclonal antibody against APC (clone CC-1; catalog numbers: OP80, Merck Chemicals GmbH, Darmstadt, Germany), and relevant fluorescence-conjugated second antibodies. Under 20× or 40× objective with green fluorescence channel, we first counted and marked olig2-positive cells, and thereafter switched to red fluorescence channel to carefully identify and count CC-1 and olig2 staining-double positive cells. The cell number was adjusted by the area of white matter investigated. To better present the images on the fluorescent staining, stack images were acquired under a 40× objective with an interval of 0.5 µm between neighboring layers, processed with deconvolution and finally Z-projected with maximum intensity.

To detect expression of BDNF in various cells, the section of spinal cord was co-stained with rabbit polyclonal antibody against BDNF (catalog numbers: NBP1-46750; Novus Biologicals), and mouse monoclonal antibody against NeuN (clone A60; Merck Chemicals GmbH) or GFAP (clone GA5; Cell Signaling Technology Europe) and followed by an incubation with relevant fluorescence-conjugated second antibodies. As described in the last paragraph, the co-localization of BDNF and GFAP staining was stack-imaged, deconvoluted and Z-projected.

Four serial paraffin-embedded sections of spinal cords were also stained with rabbit monoclonal antibody against MOG (clone E5K6T) and mouse monoclonal antibody against neurofilament-H (clone RMdO 20) (both antibodies were from Cell Signaling Technology Europe), and fluorescence-conjugated second antibodies. To evaluate the myelination, the whole spinal cord was imaged with Microlucida (MBF Bioscience). Green fluorescence-labeled MOG was measured using Image J (https://imagej.nih.gov/ij/) with fixed thresholds for all compared animals. The percentage of myelin coverage in the white matter of anterior and lateral columns was calculated.

### Quantitative RT-PCR

Total RNA was isolated from the cervical segment of spinal cord with TRIzol and from CD4-positive T lymphocytes with RNEasy Plus Mini Kit. After reverse transcription, Taqman® gene expression assays (Thermo Fisher Scientific) were used to detect the transcripts of *tumor necrosis factor* (*tnf-α*), *interleukin-1β* (*il-1β*), *chemokine (C-C motif) ligand 2* (*ccl-2*), *C-X-C motif chemokine ligand 10* (*cxcl-10*), *il-10*, *il-17a*, *ifn-γ*, and *glyceraldehyde 3-phosphate dehydrogenase* (*gapdh*). The transcription of *bdnf*, *mog*, *plp mbp*, and *eptidyl-prolyl cis-trans isomerase A* (*ppia*) was evaluated using the SYBR green binding technique with the following pairs of primers: *bdnf*, 5’-GTAAACGTCCACGGACAAGG-3’ and 5’- ATGTCGTCGTCAGACCTCTC-3’; *mog*, 5’-GAGCAAGCACCTGAATACCG-3’ and 5’-ACTCCATTGCTGCCTCTTCT-3’; *plp*, 5’-GCTGTCAGGCAGATCTTTGG-3’ and 5’-GAGCTTGATGTTGGCCTCTG-3’; *mbp*, 5’-GGCTGGAAAGAAGAGAAGCG-3’ and 5’-GTCCCATTGTTCTGGATCGC-3’; and *ppia*, 5’-AGCATACAGGTCCTGGCATCTTGT-3’ and 5’-CAAAGACCACATGCTTGCCATCCA-3’. The quantitative PCR was performed with the 7500 Fast Real-Time PCR System (Thermo Fisher Scientific) according to our established protocol (32).

### Statistics

Data were presented as mean ± SEM. For multiple comparisons, one-way or two-way ANOVA followed by Bonferroni, Tukey, or Dunnett T3 *post hoc* test (dependent on the result of Levene’s test to determine the equality of variances) were used. Two independent-samples Students *t*-test was used to compare means for two groups of cases. The correlation between MOG-positive area and APP-positive spheroids was analyzed using Pearson correlation test. To compare the effects of anti-CD52 treatment on the reduction of CD3 or Iba1-positive cells in BDNF-wildtype and deficient EAE mice, we calculated the ratios of cell density of all CD52 antibody-treated mice relative to each PBS-treated mouse. The mean ratio of CD52 antibody-treated EAE mice relative to each PBS-treated EAE mouse in BDNF-deficient and wildtype groups was determined. Thereafter, mean ratios from BDNF-wildtype and deficient groups were compared by *t*-test. All statistical analyses were performed with GraphPad Prism 8 version 8.0.2. for Windows (GraphPad Software, San Diego, CA, USA). Statistical significance was set at *p* < 0.05.

## Results

### Treatment With CD52 Antibody Depletes CD4-Positive T Lymphocytes in the Blood and Spinal Cord of C57BL/6J EAE Mice

**Figure 1:**
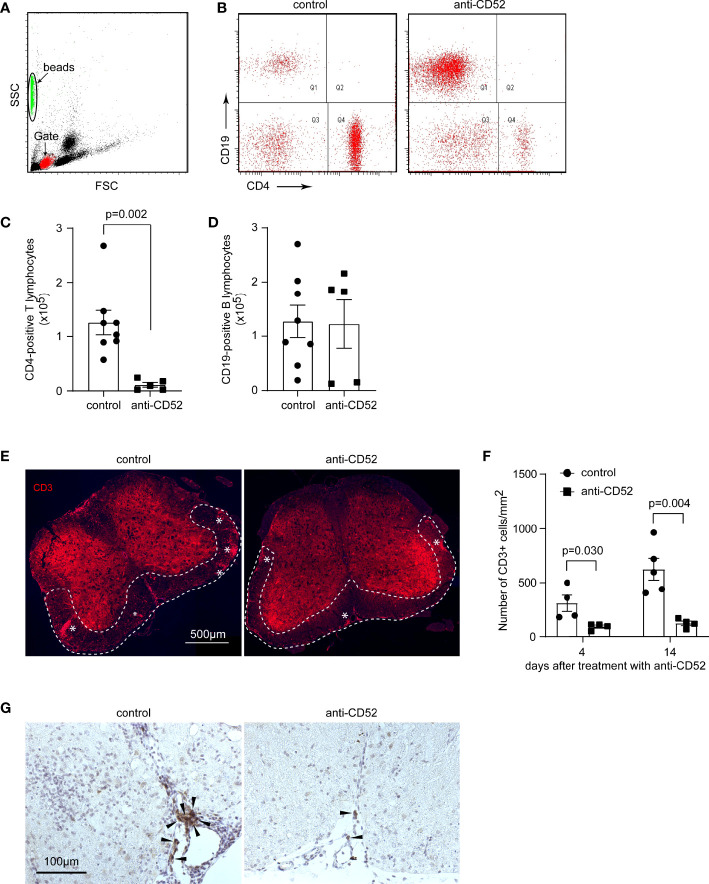
Treatment with CD52 antibody depletes T lymphocytes in the blood and spinal cord of C57BL/6J EAE mice. C57BL/6J EAE mice were treated with anti-CD52 antibodies at the peak of disease and counted 14 days later with flow cytometer for CD4- and CD19-positive lymphocytes in the blood. Treatments with CD52 antibodies significantly deleted CD4-positive cells in the following 14 days [**(A–C)**; *t* test; t (11) = 3.936; *n* ≥ 5 per group]. The number of CD19-positive B lymphocytes was not different between CD52 antibody and PBS-treated EAE mice [**(A, B, D)**; *t* test; t (11) = 0.096, *n* ≥ 5 per group]. The lumber segments of spinal cords collected from C57BL/6J EAE mice 4 and 14 days after treatments were stained with fluorescence-conjugated CD3 antibodies or immunohistochemistry with CD19 antibody. The clustering of CD3-positive lymphocytes in red is indicated with “*” [**(E)**; 14 days after treatments]. The number of CD3-positive lymphocytes in the white matter of anterior and lateral columns was significantly reduced by treatments with CD52 antibodies compared to PBS-treated control EAE mice [**(F)**; *t* test; t (6) = 2.819 and t (7) = 4.280 by 4 and 14 days after treatments, respectively; *n* ≥ 4 per group]. CD19-positive B cells in brown (marked with arrow heads) are restricted to the meninges, clustered with other immune cells in PBS-treated EAE mice, and discreetly distributed with much fewer numbers in CD52 antibody-treated EAE mice **(G)**.

To investigate the therapeutic effects of anti-CD52 in EAE mice, we first counted absolute numbers of CD4 and CD19-positive lymphocytes with flow cytometry in the blood of C57BL/6J EAE mice 14 days after treatments with anti-CD52. We observed that treatments with CD52 antibodies significantly decreased CD4-positive T lymphocytes (from 12.58 ± 2.25 ×10^4^ to 1.13 ± 0.43 ×10^4^) (**Figures 1A–C**; *t* test, *p* < 0.001); however, the number of CD19-positive B lymphocytes was not significantly changed (**Figures 1A, B, D**; *t* test, *p* > 0.05). It agreed with a previous observation that anti-CD52 treatment did not decrease CD19-positive cells in the spleen (19).

We also stained CD3 for T lymphocytes and CD19 for B cells in the spinal cord of C57BL/6J EAE mice. As shown in **Figures 1E, F**, treatment with CD52 antibodies reduced the number of CD3-positive cells 4 and 14 days after treatments in the white matter of the anterior horn in the lumbar segment by 70% and 80%, respectively, compared to PBS-treated control mice (*t* test, *p* < 0.05). CD19-positive B cells were almost exclusively restricted to the meninges, often in clusters with other immune cells in PBS-treated EAE mice, but discreetly distributed with very few numbers in CD52 antibody-treated EAE mice (**Figure 1G**), which suggests that the distribution of B lymphocytes in the spinal cord and blood might be differently regulated by the treatment of CD52 antibody.

### Treatment With CD52 Antibody Reduces Symptoms, Protects Neurons and Prevents Myelin Loss in C57BL/6J EAE Mice During the Recovering Phase

**Figure 2:**
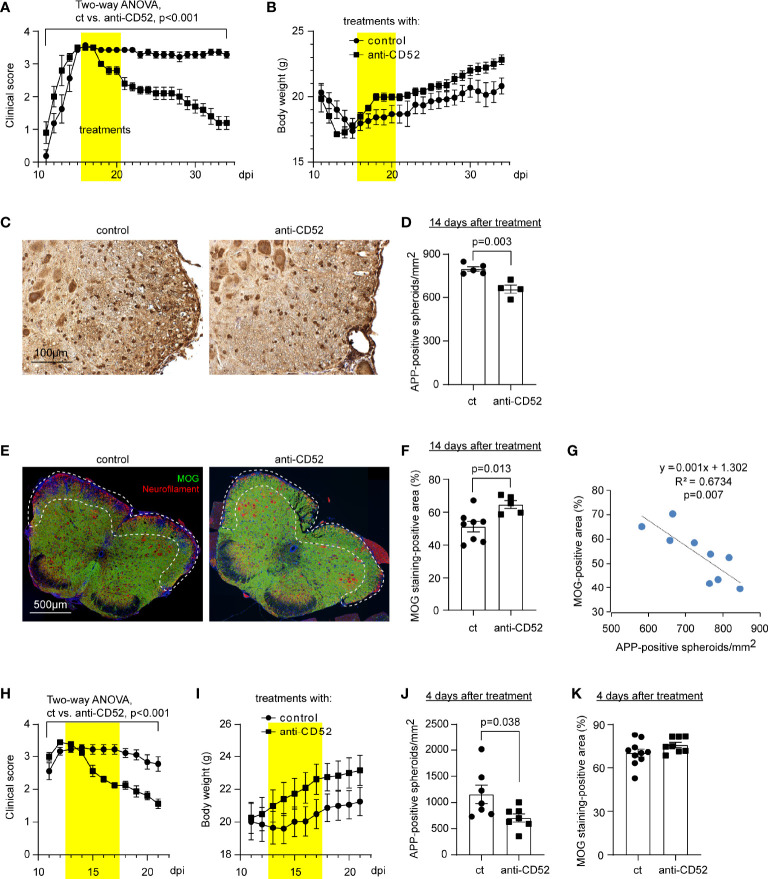
Treatment with CD52 antibody improves symptoms and pathological changes of C57BL/6J EAE mice. EAE mice established by vaccinating C57BL/6J mice with MOG35-55 were treated with CD52 antibodies or PBS at the peak of disease (~ 16 dpi). Treatments with anti-CD52 significantly attenuated clinical scores of EAE mice [**(A)** two-way ANOVA, F (1, 11) = 33.07; *n* ≥ 5 per group], and increased the body weight, although it was not statistically significant [**(B)** two-way ANOVA, F (1, 11) = 2.392; n ≥ 5 per group]. Two weeks after treatments, EAE mice were analyzed for axonal degeneration and myelin loss. Treatments with CD52 antibodies significantly reduced APP-positive spheroids (in brown) [**(C, D)** *t* test, t (7) = 4.485; *n* ≥ 4 per group], and markedly increased the coverage of MOG-positive myelin (in green) [**(E, F)** *t* test, t (11) = 2.978; *n* ≥ 5 per group] in the white matter of anterior and lateral columns at the lumber spinal cord (as shown in E with the frame), compared with PBS-treated EAE mice. The presented images are from EAE mice 14 days post treatments. Interestingly, the number of APP-positive spheroids was negatively correlated with the area of MOG-positive myelin [**(G)** Pearson correlation test; *n* = 9]. EAE mice were also analyzed within 4 days after treatments. Anti-CD52 treatment immediately reduced the clinical scores of EAE mice [**(H)** two-way ANOVA, F (1, 15) = 17.24; *n* ≥ 8 per group], and increased the body weight, although not statistically significant [**(I)** two-way ANOVA, F (1, 15) = 1.515; n ≥ 8 per group]. Histological analysis showed that treatments with anti-CD52 antibodies significantly decreased the number of APP-positive spheroids [**(J)** *t* test, t (12) = 2.339; *n* = 7 per group] but did not change the coverage of MOG-positive myelin in the white matter of lumber spinal cord [**(K)** *t* test, t (16) = 1.581; *n* ≥ 8 per group].

After we observed that treatment with CD52 antibody dramatically decreased T lymphocytes in EAE mice, we examined effects of anti-CD52 therapy on clinical symptoms and pathological changes in the spinal cord. We treated C57BL/6J EAE mice with CD52 antibodies at the peak of disease (around 16 dpi) and monitored EAE symptoms for 3 weeks until around 35 dpi. We observed that treatments with CD52 antibodies quickly and significantly attenuated clinical scores of EAE as compared with PBS treatments (**Figure 2A**; two-way ANOVA, *p* < 0.001). Accordingly, the body weight was increased by anti-CD52 treatments although it was not significantly changed (**Figure 2B**; two-way ANOVA, *p* = 0.150).

Fourteen days after the 5-day treatment of CD52 antibody, the spinal cord of EAE mice was evaluated for axonal dysfunction and demyelination by staining tissues with anti-APP and MOG antibodies, respectively. We observed that treatments with CD52 antibodies compared to PBS treatments significantly decreased the number of APP-positive spheroids (**Figures 2C, D**; *t* test, *p* = 0.003) and increased MOG-positive myelin (**Figures 2E, F**; *t* test, *p* = 0.004) in the white matter of anterior and lateral columns in the lumber segment of spinal cord. The decreased number of APP-positive spheroids was negatively correlated with the increased coverage of myelin (**Figure 2G**; Pearson correlation test, *p* = 0.007). Thus, treatments with CD52 antibodies protect neurons and prevent myelin loss during the recovering phase of EAE mice.

In further experiments, we analyzed C57BL/6J EAE mice within 4 days after the 5-day anti-CD52 treatments. As shown in **Figures 2H, I**, the improvement of symptoms of EAE mice already started during the treatment of anti-CD52 antibody (two-way ANOVA, *p* < 0.001). In pathological examinations, treatments of anti-CD52 antibody reduced APP-positive spheroids (**Figure 2J**; *t* test, *p* = 0.038), but did not change the coverage of MOG-positive myelin (**Figure 2K**; *t* test, *p* = 0.134), which indicates that treatments with anti-CD52 serve immediate neuroprotective effects in EAE models, before the damaged myelin sheath is improved.

### Treatment With CD52 Antibody Improves Symptoms of SJL EAE Mice During the Recovering Phase

**Figure 3:**
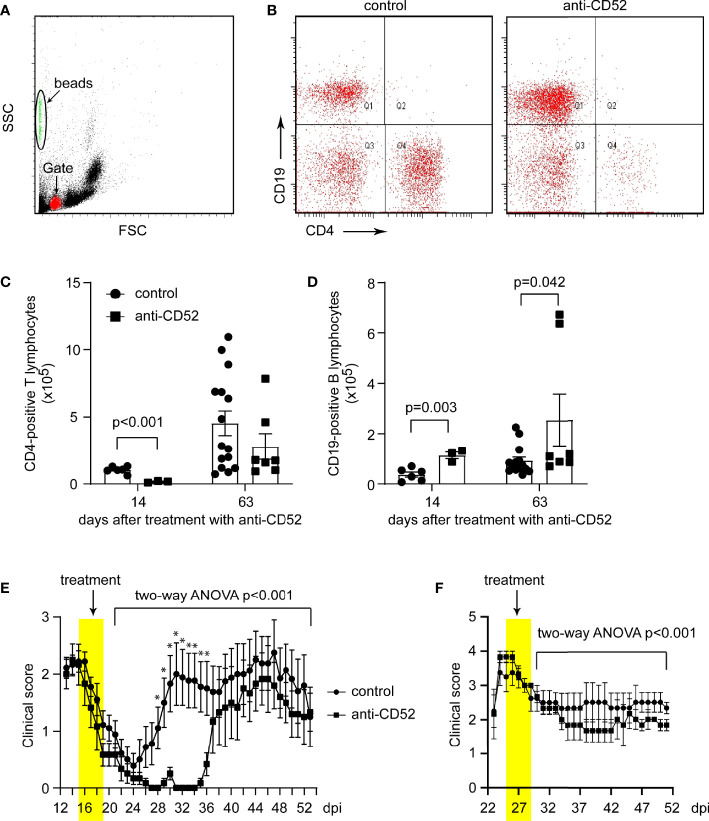
Treatment with CD52 antibody depletes CD4-positive T lymphocytes and inhibits symptoms of SJL EAE mice. Eight-week-old SJL mice were immunized with PLP139-153 in a complete Freund’s adjuvant to develop EAE. At the peak of disease, paired EAE mice with similar symptoms were treated with CD52 antibodies and PBS for 5 consecutive days. Early-onset SJL EAE mice were counted with flow cytometer for CD4- and CD19-positive lymphocytes in the blood 14 and 63 days post anti-CD52 treatment. Treatments with CD52 antibodies significantly deleted CD4-positive cells in the following 14 days [**(A–C)** *t* test; t (7) = 5.840; *n* ≥ 3 per group]. Thereafter, CD4 cell population was regenerated as the number of CD4-positive lymphocytes in CD52 antibody-treated mice was no longer different from that of PBS-treated EAE mice on 63 days after treatments [**(C)** *t* test; t (20) = 1.165, *p* = 0.258; *n* ≥ 7 per group]. The numbers of CD19-positive B lymphocytes were increased 14 and 63 days after treatments with CD52 antibodies compared with PBS-treated control EAE mice [**(A, B, D)** *t* test; by 14 days, t (7) = 4.507, *n* ≥ 3 per group and by 63 days, t (19) = 2.182, *n* ≥ 7 per group]. In early-onset EAE mice, treatments with anti-CD52 significantly delayed EAE relapse **(E)**, two-way ANOVA analysis showing the effect of anti-CD52 therapy from 1 to 34 days post treatments, F(1,67) = 48.931; *: *t* test comparing two EAE groups on individual days, *p* < 0.05; *n* = 6 and 9 for anti-CD52 and PBS control groups, respectively). In late-onset EAE mice, treatments with anti-CD52 significantly attenuated EAE severity (**F**, two-way ANOVA analysis showing the effect of anti-CD52 therapy from 1 to 21 days post treatment, F(1,41) = 19.105; *n* = 3 and 5 for anti-CD52 and PBS control groups, respectively).

To verify the therapeutic effects of anti-CD52 antibodies, we examined our second EAE model on SJL mice, a typical animal model for remission-relapse MS. We observed two different types of EAE with symptoms appearing before and after 21 dpi. We named these two EAE models as early-onset EAE and late-onset EAE. In the early-onset EAE, EAE symptoms appeared around 12 dpi, reached a peak around 14 dpi, and substantially recovered thereafter, which was followed by a relapse in 2 weeks. We treated EAE mice with anti-CD52 from 15 dpi for 5 days. We counted the numbers of CD4 and CD19-positive lymphocytes with flow cytometry in the blood of early-onset SJL EAE mice 14 and 63 days after treatments with anti-CD52. We observed that treatments with CD52 antibodies significantly decreased CD4-positive T lymphocytes (from 10.57 ± 1.04 ×10^4^ to 1.62 ± 0.33 ×10^4^) in the following 14 days (**Figure 3A–C**; *t* test, *p* < 0.001). By 63 days, the number of CD4-positive lymphocytes in anti-CD52-treated EAE mice was not different from that of PBS-treated control EAE mice (**Figures 3A–C**; *t* test, *p* > 0.05), which indicates that CD4-positive cell population was regenerated in the blood. Surprisingly, by 14 and 63 days, the numbers of CD19-positive B lymphocytes were significantly increased by anti-CD52 treatments as compared with PBS treatments (**Figures 3A, B, D**; *t* test, *p* = 0.003 by 14 days, and 0.042 by 63 days), which was different from two previous observations that anti-CD52 treatments depleted both T and B lymphocytes in the blood or spleen (18, 20).

Treatments with anti-CD52 starting at 15 dpi for 5 days did not stop EAE relapse; however, it significantly delayed EAE relapse (**Figure 3E**; two-way ANOVA, *p* < 0.001). The severity of EAE in the relapse was not significantly different between EAE mice treated with CD52 antibodies and PBS.

In the late-onset EAE, EAE symptoms appeared after 21 dpi and reached the peak 3 days later. For these EAE mice, there was a mild recovery, and the severe symptom (~3 points of score) during the recovering phase remained constant for at least 3 weeks. Treatments with CD52 antibodies starting from the peak of disease for 5 days significantly attenuated the severity of EAE symptoms as compared with PBS treatments (**Figure 3F**; two-way ANOVA, *p* < 0.001). The experiments on SJL EAE mice suggest that treatments with CD52 antibodies improve the recovery of EAE mice.

### Treatment With CD52 Antibody Attenuates Inflammatory Activation in the Spinal Cord of C57BL/6J EAE Mice

**Figure 4:**
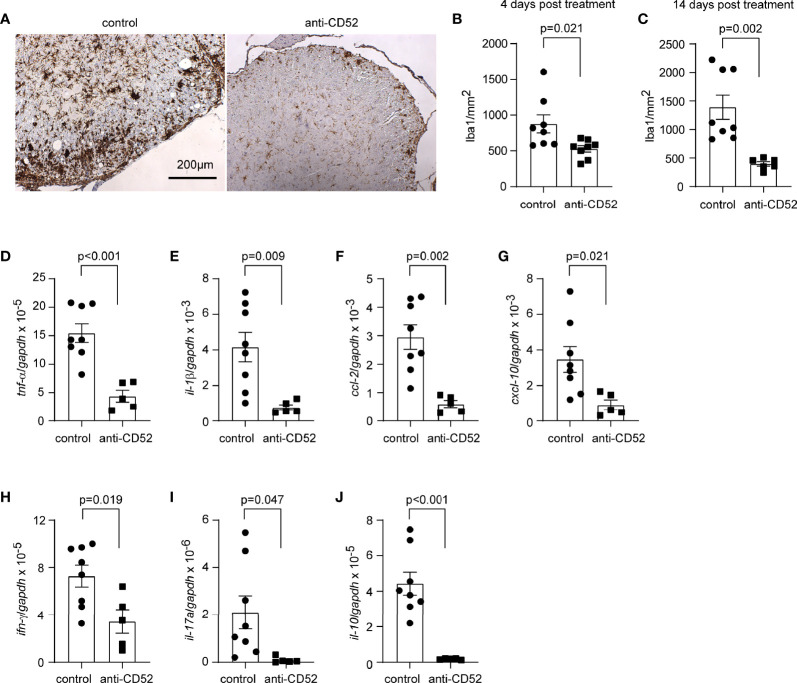
Treatment with CD52 antibody decreases inflammatory activation in the spinal cord of C57BL/6J EAE mice. C57BL/6J EAE mice were treated with anti-CD52 antibodies at the peak of disease. Four and 14 days after treatments, the spinal cord was isolated from EAE mice for the analysis of Iba1-positive cell infiltration and inflammatory gene transcription. Treatment with CD52 antibodies decreased the number of Iba1-positive cells (in brown) in the white matter of anterior and lateral columns at the lumber spinal cord compared with PBS-treated EAE mice 4 and 14 days after treatments **(A–C)** *t* test; t (14) = 2.608 and t (12) = 3.951 for 4 and 14 days, respectively; *n* ≥ 6 per group; The images are from EAE mice 14 days post treatments). Similarly, treatments with anti-CD52 antibodies strongly decreased the transcription of both pro- and anti-inflammatory genes **(D–J)** *t* test; t (11) = 4.903, 3.181, 4.176, 2.681, 5.120, 2.739 and 2.233 for *tnf-α*, *il-1β*, *ccl-2*, *cxcl-10*, *il-10*, *ifn-γ* and *il-17a*, respectively; *n* ≥ 5 per group).

It is known that the focal lymphocytic infiltration leads to inflammatory damage of myelin and axons (33). We continued to examine the inflammatory activation of CD52 antibody-treated C57BL/6J EAE mice. As shown in **Figures 4A–C**, treatments with CD52 antibodies compared with PBS treatments significantly reduced the number of Iba1-positive microglia and macrophages in the white matter of anterior horn in the lumber spinal cord 4 and 14 days after anti-CD52 treatments (*t* test; *p* < 0.05). When comparing EAE mice 4 and 14 days after PBS treatment, we observed that the infiltration of microglia/macrophages appeared to further increase after the EAE score reached the peak, since the number of Iba1-positive cells on 14 days post treatments (1393.0 ± 213.8/mm^2^) was more than the cell number on 4 days after treatments (877.6 ± 126.1/mm^2^), although the increase of number was not statistically significant (t test, t (14) = 2.075; *p* = 0.057).

We also measured inflammatory gene transcripts in the cervical segment of spinal cord. We observed that treatments with anti-CD52 antibodies compared to PBS strongly down-regulated the transcription of various inflammatory genes, e.g., *tnf-α*, *il-1β*, *ccl-2*, and *cxcl-10*, and the marker genes (*ifn-γ*, *il-17a*, and *il-10*) of both encephalitogenic (Th1 and Th17 cells) and regulatory T lymphocytes cells 14 days after treatments (**Figures 4D–J**; *t* test; *p* < 0.05). However, the transcription of all tested genes was not significantly changed 4 days after anti-CD52 treatments (data not shown).

### Treatment With CD52 Antibody Has a Potential to Promote Re-Myelination in the Spinal Cord of C57BL/6J EAE Mice

**Figure 5:**
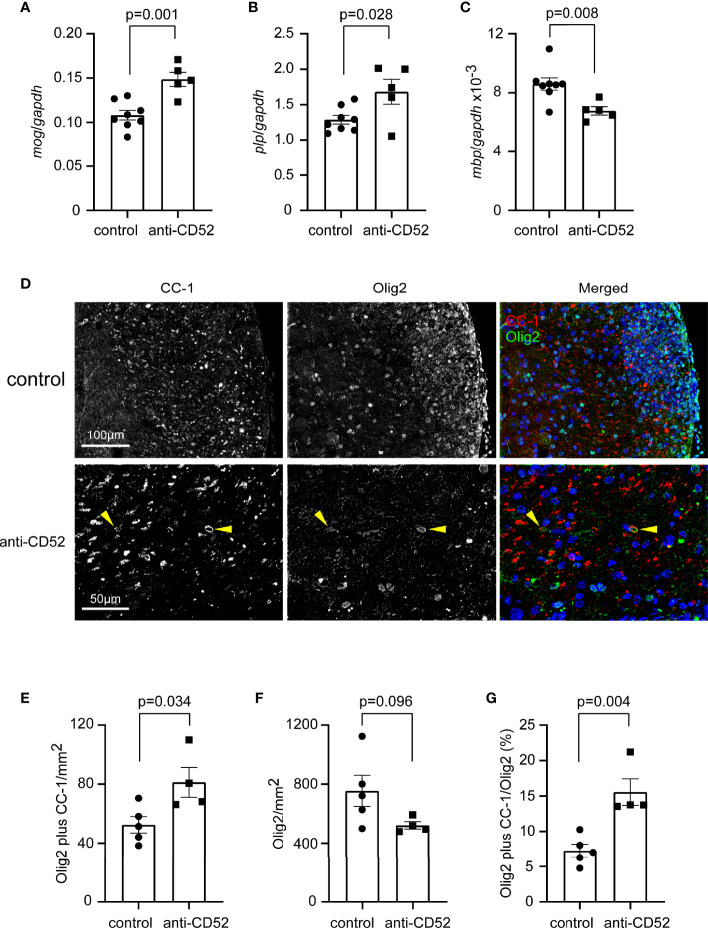
Treatment with CD52 antibody has a potential to promote remyelination in the spinal cord of C57BL/6J EAE mice. C57BL/6J EAE mice were treated with CD52 antibodies and PBS at the peak of disease. Two weeks later, the spinal cord was isolated for analysis of transcription of myelin protein genes, *mog*, *plp* and *mbp* [**(A–C)** *t* test; t (11) = 4.378, 2.526 and 3.208 for *mog*, *plp* and *mbp*, respectively; *n* ≥ 5 per group] and for the immunohistological analysis of olig2 and CC-1-positive oligodendrocytes. The olig2 and CC-1 double positive oligodendrocytes are indicated with arrow heads **(D)**. Treatments with CD52 antibodies significantly increased the density of olig2/CC-1-positive oligodendrocytes [**(E)** *t* test; t (7) = 2.636; *n* ≥ 4 per group] and tended to decrease the number of single olig2-positive cells [**(F)** *t* test; t (7) = 1.926; *n* ≥ 4 per group]. The percentage of olig2/CC-1-double positive cells among total olig2-positive cells was strongly increased by anti-CD52 therapy [**(G)** *t* test; t (7) = 4.252; *n* ≥ 4 per group].

In our study, EAE mice were treated with CD52 antibodies at the peak disease stage. The improved symptoms and myelination in the spinal cord might be associated with a promoted re-myelination. We detected transcription of genes encoding myelin proteins in the cervical segment of spinal cord of EAE mice 14 days after treatments and observed that anti-CD52 treatment significantly up-regulated the transcription of *mog* and *plp* genes compared with PBS injection (**Figures 5A, B**; *t* test; *p* < 0.05), although the transcription of *mbp* was significantly decreased (**Figure 5C**; *t* test; *p* < 0.05). It should be noted that the transcriptional level of *mog* and *plp* genes was > 100 folds higher than that of *mbp* gene.

We co-stained the spinal cord with olig2 antibody and mouse clonal antibody (clone CC-1) against APC, as shown in published studies (20), and calculated the densities of olig2+CC-1+ maturated oligodendrocytes, which has been used as a surrogate readout of remyelination (34). We observed that olig2-positive cells accumulated at EAE lesion sites (**Figures 5D, F**); however, were rarely co-stained by CC-1 antibody. Treatments with CD52 antibodies significantly increased the densities of olig2/CC-1- double immunoreactive cells in the spinal cord (**Figures 5D, E**; *t* test, *p* < 0.05). Moreover, we showed that the percentage of olig2/CC-1- double cells among total olig2-positive cells was significantly higher in CD52 antibody-treated EAE mice than in PBS-treated EAE mice (**Figure 5G**; *t* test, *p* < 0.05). Our experiments suggested that anti-CD52 treatments potentially promote maturation of OPCs and subsequent re-myelination in the spinal cord of EAE mice.

### Neuronal BDNF Regulates the Neuroprotective Effects of Anti-CD52 Therapy in C57BL6 EAE Mice

BDNF deficiency in the CNS has been shown to exacerbate EAE pathology (24); however, it remains unclear which CNS cell type contributes BDNF that prevents the progress of EAE. In this project, we conditionally knocked out BDNF expression in neurons, astrocytes, microglia or CD4-positive lymphocytes and then established EAE models with these mice. We observed that deletion of BDNF in neurons exacerbated EAE symptoms (**Supplementary Figure 1A**; two-way ANOVA, *p* < 0.05), while deletion of BDNF in astrocytes (**Supplementary Figure 1C**; two-way ANOVA, *p* < 0.05) or in CD4-postive T lymphocytes (**Supplementary Figure 2D**; two-way ANOVA, *p* < 0.05) protected mice from developing EAE symptoms. Deletion of BDNF in microglia did not alter EAE symptoms (data not shown), which was in line with a previous observation that BDNF-deficient bone marrow reconstruction did not change EAE symptoms in the whole body-irradiated recipient mice (24). Our previous study showed that the whole-body irradiation allowed the reconstructed bone marrow cells to enter the brain and create a BDNF-deficient population of brain macrophages (35). Since BDNF from neurons prevents EAE progression, whereas BDNF from astrocytes and T lymphocytes promotes EAE development, we chose *bdnf^fl/fl^/camk2α-cre* ^tg^ and *bdnf^fl/fl^/camk2α-cre* ^wt^ mice to investigate the role of BDNF in anti-CD52 therapy.

**Figure 6:**
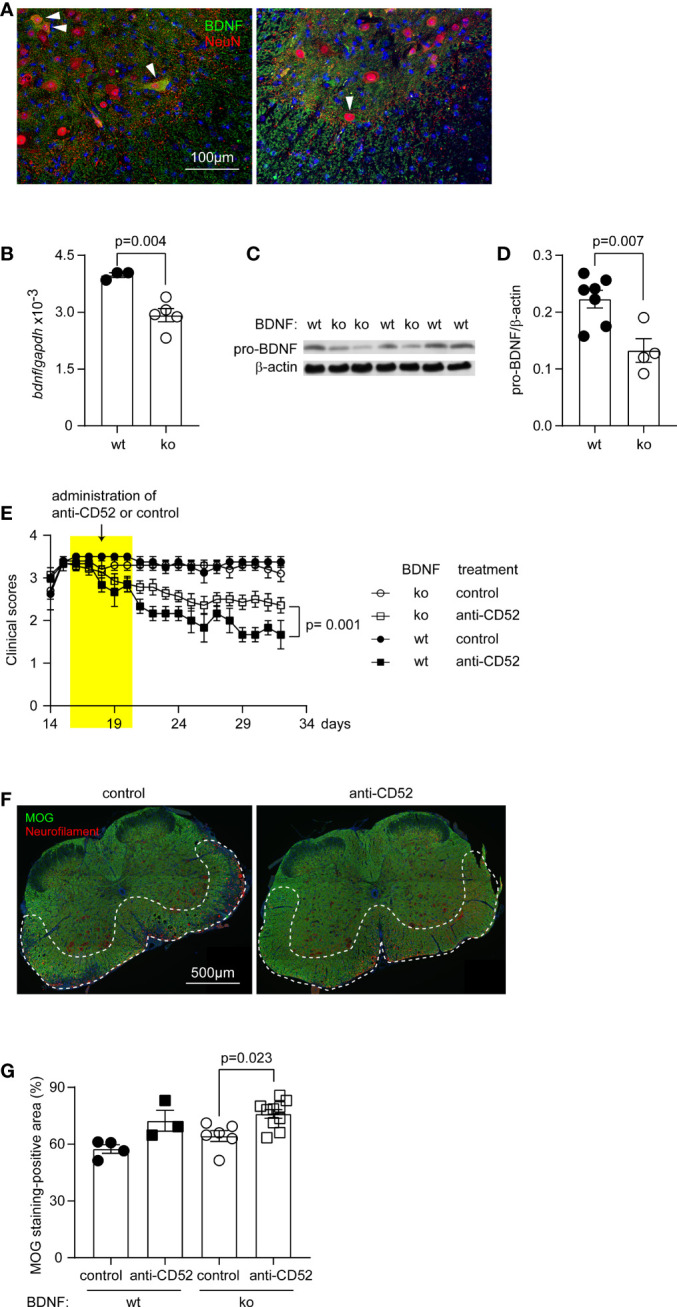
Treatment with CD52 antibody improves symptoms and pathology in both neuronal BDNF-deficient and wildtype EAE mice. Eight-week-old C57BL/6 littermate mice with (ko) and without (wt) BDNF deficiency in neurons were immunized with MOG35-55 in a complete Freund’s adjuvant. The reduction of BDNF expression in neurons of spinal cord was detected by immunological staining of BDNF and NeuN [**(A)** BDNF-expressing neurons are marked with arrow heads] and quantitated by real-time PCR measurement of *bdnf* gene transcripts in the cervical spinal cord [**(B)** *t* test; t (6) = 4.444; *n* ≥ 3 per group], and Western blot evaluation of pro-BDNF in the brain homogenate **(C, D)** *t* test; t (9) = 3.483; *n* ≥ 4 per group). At the peak of disease (around 16 dpi), CD52 antibodies were subcutaneously administered for 5 days. PBS was injected as a control. Clinical symptoms were monitored for around 2 weeks after anti-CD52 treatments. The clinical scores were not significantly different between BDNF wt and ko mice after injection with PBS [**(E)** two-way ANOVA followed by Bonferroni *post-hoc* test, *p* = 0.718]; whereas the clinical scores of both BDNF wt and ko mice were significantly reduced by treatments with CD52 antibodies as compared with EAE mice receiving PBS injection [**(E)** two-way ANOVA followed by Bonferroni *post-hoc* test; F (3, 15) = 11.02; *n* ≥ 3 per group]. Interestingly, the anti-CD52 treatments-induced recovering of BDNF-wt EAE mice was significantly better than of BDNF-ko EAE mice [**(E)** two-way ANOVA followed by Bonferroni *post-hoc* test, *p* = 0.001]. Myelination in BDNF-ko EAE mice were further evaluated by immunofluorescent staining of MOG **(F)**. Treatments with CD52 antibodies compared with PBS treatments significantly increased the coverage of MOG-positive myelin (in green) in the white matter of anterior horn of the lumber spinal cord (as shown with the frame) **(G)** one-way ANOVA followed by Bonferroni *post-hoc* test; F (3, 19) = 7.858; *n* ≥ 3 per group).

By histological analysis, we observed that not all NeuN-positive cells (representing neurons) of wild-type (*bdnf^fl/fl^/camk2α-cre* ^wt^) mice expressed BDNF in the anterior horn of spinal cord; and there were a few neurons in BDNF-deficient (*bdnf^fl/fl^/camk2α-cre* ^tg^) mice expressing BDNF (**Figure 6A**). However, quantitative PCR showed that the transcriptional level of *bdnf* gene in the cervical spinal cord of *bdnf^fl/fl^/camk2α-cre* ^tg^ EAE mice was significantly lower than that in *bdnf^fl/fl^/camk2α-cre* ^wt^ EAE mice (**Figure 6B**; *bdnf*/*gapdh*: 2.920 ± 0.175 ×10^-3^ vs. 3.977 ± 0.006 ×10^-3^; t test, *p* < 0.05). Quantitative Western blot indicated the reduction of pro-BDNF by ~50% in the brain homogenate of *bdnf^fl/fl^/camk2α-cre* ^tg^ mice compared with *bdnf^fl/fl^/camk2α-cre* ^wt^ mice (**Figures 6C, D**; pro-BDNF/β-actin: 0.663 ± 0.104 and 1.115 ± 0.078 in *bdnf^fl/fl^/camk2α-cre* ^tg^ and *bdnf^fl/fl^/camk2α-cre* ^wt^ mice, respectively; t test, *p* < 0.05). Our results corroborated a previous observation that c*amk2α* promoter-driven Cre expression recombines genes in neurons in the spinal cord and modifies EAE pathogenesis (36).

We chose EAE mice with similar clinical scores at the peak of disease and injected mice with CD52 antibody or PBS. In the recovering phase, EAE littermate mice with and without deficiency of BDNF in neurons displayed similar clinical scores after receiving PBS injection (**Figure 6E**; two-way ANOVA followed by Bonferroni *post-hoc* test, *p* = 0.718). Treatment with CD52 antibody significantly reduced the clinical scores in both EAE mice with and without BDNF deficiency; interestingly, the clinical improvement of BDNF-wildtype EAE mice, as demonstrated by the decrease in clinical scores, was more pronounced than that of BDNF-deficient EAE mice (**Figure 6E**; two-way ANOVA followed by Bonferroni *post-hoc* test, *p* < 0.001).

Myelination in BDNF-deficient and wildtype EAE mice was analyzed by staining MOG in the spinal cord (**Figure 6F**). Treatment with anti-CD52 antibody compared with PBS treatment significantly increased the coverage of MOG-positive myelin in the white matter of anterior horn of lumbar spinal cord in neuronal BDNF-deficient EAE mice (**Figure 6G**; one-way ANOVA followed by Bonferroni *post-hoc* test, *p* < 0.05), which was comparable to the effect of anti-CD52 treatment on the myelination in BDNF-wildtype littermate EAE mice and C57BL/6J EAE mice (**Figures 2E, F**).

### Neuronal BDNF Regulates the Effects of Anti-CD52 Therapy on Inflammatory Infiltration in C57B6 EAE Mice

**Figure 7:**
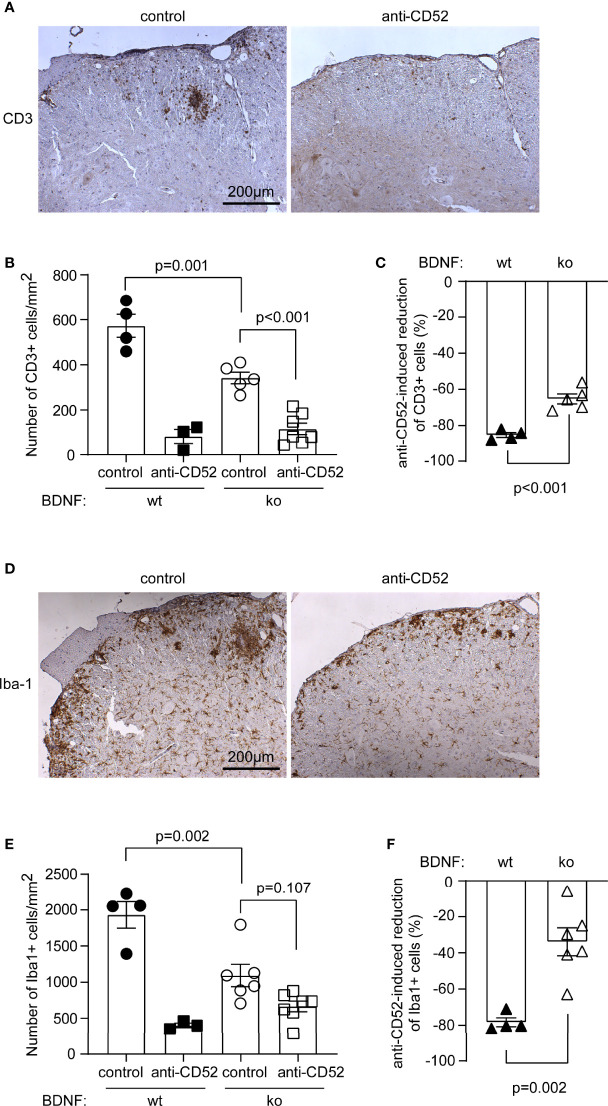
Deficiency of neuronal BDNF attenuates CD52 antibody-induced reduction of inflammatory infiltrates in EAE mice. Eight-week-old C57BL/6 littermate mice with (ko) and without (wt) BDNF deficiency in neurons were immunized with MOG35-55 in a complete Freund’s adjuvant. EAE mice were administered subcutaneously with CD52 antibodies or PBS for 5 days at the peak of disease. Two weeks after treatments, the lumber segment of spinal cord was analyzed with immunohistochemistry for the infiltration of T cells and microglia/macrophages **(A, D)** immune reactive cells are labelled in brown). Treatments of CD52 antibodies significantly reduced the number of CD3-positive cells **(B)** one-way ANOVA followed by Bonferroni *post-hoc* test; F (3, 15) = 42.81; *n* ≥ 3 per group) and tended to decrease Iba1-positive cells **(E)** one-way ANOVA followed by Bonferroni *post-hoc* test; F (3, 16) = 21.20; *n* ≥ 3 per group) in BDNF-ko EAE mice. Interestingly, deficiency of neuronal BDNF significantly decreased the number of both CD3 and Iba1-positive cells in the lumber spinal cord of PBS-treated EAE mice **(B, E)**. BDNF deficiency also significantly attenuated CD52 antibody-induced reduction of CD3- and Iba1-positive cells relative to PBS-treated EAE mice **(C, F)** *t* test; t (7) = 6.014 and t (8) = 4.496 for CD3 and Iba1-positive cells, respectively; *n* ≥ 4 per group).

After observing therapeutic effects of anti-CD52 antibody in BDNF-deficient EAE mice, we analyzed how neuronal BDNF and anti-CD52 treatments regulated the inflammatory activation in EAE. It was the same as in BDNF-wildtype EAE mice, treatment with anti-CD52 antibody compared to PBS treatment significantly reduced the number of CD3-positive T lymphocytes in the anterior horn of lumber spinal cord in BDNF-deficient EAE mice (**Figures 7A, B**; one-way ANOVA, *p* < 0.05). However, anti-CD52 therapy did not significantly reduce the number of Iba1-positive microglia/macrophages in BDNF-deficient EAE mice (**Figure 7D**, E; one-way ANOVA, *p* > 0.05).

BDNF deficiency in neurons decreased the number of both CD3-positive cells and Iba1-positive cells in PBS-treated EAE mice (**Figure 7B, E**; one-way ANOVA, *p* < 0.05), which suggests that neuronal BDNF promotes the infiltration of immune cells in the lesion sites of EAE mice. To evaluate the effect of BDNF on anti-CD52 treatment-induced reduction of CD3 or Iba1-positive cells, we calculated the difference of cell density between CD52 antibody and PBS-treated EAE mice that were deficient or wild-type in neuronal BDNF. As shown in **Figure 7C, F**, deficiency of neuronal BDNF significantly attenuated CD52 antibody-induced reduction of CD3-positive lymphocytes and Iba1-positive microglia/macrophages (*t* test, *p* < 0.05).

## Discussion

MS is a T- and B-lymphocytes-mediated chronic inflammatory disease that results in demyelination and neuronal loss in the CNS (1). The list of drugs that modulate immune activity has grown in recent years. Their effects on remyelination and neurodegeneration are also gaining increasing attention. We treated EAE mice with CD52 antibodies at the peak of clinical score, which was aimed to investigate the effects of anti-CD52 therapy on the recovering of EAE mice. Treatment with CD52 antibody: 1) deleted T lymphocytes but not B cells in the blood; 2) inhibited inflammatory activation, protected neurons and potentially promoted remyelination in the spinal cord; and 3) attenuated symptoms of EAE mice. We also observed that BDNF deficiency in neurons attenuated the effect of anti-CD52 treatment on reducing clinical scores and neuroinflammation in EAE mice; however, did not negate the therapeutic effects of the CD52 antibody.

Administration of CD52 antibodies at the peak of disease reduced the clinical score and the number of APP-positive spheroids in the spinal cord of EAE mice within 4 days, without affecting myelin coverage, demonstrating the immediate neuroprotective effects of CD52 antibody. CD52 antibody does not directly act on neurons and microglia (21). This beneficial effect should come from the T cell depletion-induced inhibition of microglia/macrophages-involved inflammatory activation. It has been observed that inflammatory activation causes the energy crisis of neurons in EAE mice (4, 5).

In a later phase (e.g. 14 days after treatment), anti-CD52 therapy adds protective effects on myelination. We observed that the inflammatory activation and demyelination became severer after the peak of clinical score in our EAE mice. The reduction of myelin loss in CD52 antibodies-treated EAE mice might be due to the decrease of demyelination or increase of remyelination or both. It is not surprising that inflammatory suppression prevents demyelination (37). Our previous study showed that deficiency of inhibitor of NF-κB kinase subunit β in myeloid cells reduces inflammatory activation and prevents demyelination in the spinal cord of EAE mice (30). The shift from classic to alternative inflammatory activation in the spinal cord favors remyelination (38–40). It was also reported that Th1 and Th17 T lymphocytes inhibit (7, 8), while Treg cells promote remyelination (9). In our EAE model, anti-CD52 treatment had a potential to promote remyelination, as evidenced by the increase of maturated oligodendrocytes, and transcription of *mog* and *plp* genes. However, anti-CD52 treatment decreased transcription of both pro- and anti-inflammatory genes (e.g. *tnf-α*, *il-1β*, *ccl-2*, and *cxcl-10*), as well as marker genes (*ifn-γ*, *il-17a* and *il-10*) of Th1, Th17 and Treg lymphocytes. The remyelination in anti-CD52-treated EAE mice appears to be driven by the release of inhibitory effects from pro-inflammatory activation. In a previous EAE study, anti-CD52 therapy starting at a late disease stage modified neither demyelination nor remyelination (20). A potential explanation is that the inflammatory activation in the EAE mice has been resolved and cannot be further modified by CD52 antibodies.

However, it should be noted that: 1) treatment with CD52 antibody drastically reduces microglia/macrophages, which could prevent the removal of myelin debris and the synthesis of sterol, thereby slowing down remyelination (41, 42); 2) anti-inflammatory activation (e.g. decrease of TNF-α secretion) suppresses the proliferation of OPCs (43, 44). We observed that treatment with CD52 antibodies reduced olig2-positive cells (including OPCs and mature oligodendrocytes) in EAE mice; and 3) anti-CD52 treatment increased the transcription of *mog* and *plp* genes but decreased the transcription of *mbp* gene. It is not known whether the imbalance between the expression of different myelin proteins affects the quality of regenerated myelin. Therefore, the effect of anti-CD52 therapy on remyelination needs further evaluation. Some experiments, such as quantification of G-ratios, which more directly demonstrates the regenerated myelin, should be considered.

BDNF is expressed by neurons, astrocytes and various immune cells. BDNF protects neurons and supports OPC proliferation in traumatically injured nerve tissues (45). We observed that BDNF from neurons attenuated EAE symptoms, in agreement with a previous study (24). Neuronal deficiency of BDNF weakened the effect of CD52 antibody on improving EAE symptoms. Thus, neuronal BDNF partially mediates or regulates the neuroprotective effects of anti-CD52 treatment in EAE mice. Interestingly, BDNF promoted inflammatory infiltration in the spinal cord of EAE mice. The lower level of inflammatory activation in neuronal BDNF-deficient EAE mice might be responsible for the attenuated effect of anti-CD52 therapy on the inflammatory inhibition. The transcription of *BDNF* gene in peripheral mononuclear blood cells of MS patients is upregulated in the relapsing phase relative to the remission phase (46). The pathophysiological role of BDNF in MS remains to be clarified.

We observed that deletion of BDNF in astrocytes improves EAE symptoms, which is in contrast to a previous observation that BDNF deficiency in astrocytes exacerbates EAE pathology (23). It should be noted that BDNF was deleted at adulthood in our experiments, while from embryogenesis in Linker’s study. Our experiment also showed that deletion of BDNF in CD4-positive T lymphocytes improved EAE symptoms. Interestingly, BDNF deficiency decreased *ifn-γ* transcription in CD4-positive spleen cells, suggesting that BDNF has the potential to modulate T cell differentiation. BDNF deficiency has been reported to block the maturation of CD4/CD8 double-negative thymocytes and reduce the number of CD4- or CD8-positive cells in the peripheral lymphatic organs (47).

Alemtuzumab is approved for the treatment of highly active relapsing-remitting MS. In phase III clinical trials (CARE-MS I and II), the following 4-year extension study, and a 2-year real-world study, administration of alemtuzumab (even switched from IFN-β therapy) not only reduces the relapse rate of MS and gadolinium-enhancing lesions in the CNS, but also prevents the progression of disability and loss of brain volume (13, 14, 48, 49). Together with our observations in EAE animals, anti-CD52 therapy represents an efficient immune reconstitution therapy that induces long-term remission and prevents neurodegeneration in MS patients. Unfortunately, treatment with alemtuzumab potentially causes secondary autoimmune diseases (50) that limit clinical administration of alemtuzumab with a full risk assessment and mandatory safety surveillance (51).

It should be noted that treatment with CD52 antibody increased rather than decreased CD19-positive B lymphocytes in the blood of our SJL-EAE mice. In C57BL/6J EAE mice, the number of CD19-positive B lymphocytes in the blood was not altered by CD52 antibody. Indeed, a previous study has shown that anti-CD52 treatment did not change the number of B lymphocytes in the spleen (19). The more recruitment of CD19-positive cells into the meninges of our PBS-treated C57BL/6J EAE mice than in CD52 antibody-treated EAE mice might be driven by the local and strong inflammatory activation, which could not represent the cell number of B lymphocytes in the blood. In MS patients treated with alemtuzumab, B cells repopulate much faster than T cells, returning to baseline by 3 months and rising to 165% of baseline by 12 months after treatment (52). It is unclear whether the expansion of B lymphocytes in our SJL EAE mice promotes the EAE relapse. It is more important to consider whether the rapid and/or high B cell recovery compared to T cell recovery leads to a dysregulated expansion of autoreactive B-cells in MS patients, which might underly the occurrence of secondary autoimmune diseases after alemtuzumab therapy (50).

In summary, treatment with CD52 antibody deletes T lymphocytes and dramatically inhibits inflammatory activation in the spinal cord of EAE mice, which protects neurons, potentially promotes remyelination and improves EAE symptoms. Neuronal deficiency of BDNF attenuates the effects of anti-CD52 therapy on neuroprotection and inflammatory inhibition in EAE mice. Surprisingly, treatment with CD52 antibody might increase CD19-positive B lymphocytes in the blood of SJL EAE mice. Whether the activation profile of expanded B cells is different from that of B cells before anti-CD52 treatment and whether this expansion promotes EAE relapse are the focus of our following studies. It will be also interesting to investigate whether the rapid recovering of B cells in alemtuzumab-treated MS patients drives the second autoimmune disorders.

## Data Availability Statement

The original contributions presented in the study are included in the article/**Supplementary Material**. Further inquiries can be directed to the corresponding author.

## Ethics Statement

The animal study was reviewed and approved by Landesamt für Verbraucherschutz, Saarland. Written informed consent was obtained from the owners for the participation of their animals in this study.

## Author Contributions

YL: conceptualized and designed the study, acquired funding, conducted experiments, acquired and analyzed data, and wrote the manuscript. WH and QL: conducted experiments, acquired data, and analyzed data. MM: offered animal facility and supervised animal experiments. KF: offered laboratory equipment and supervised the laboratory experiments. All authors contributed to the article and approved the submitted version.

## Funding

This work was funded by Sanofi/Genzyme (GZ-2016-11588) and Medical Faculty of Universität des Saarlandes through HOMFOR2019 (to YL). However, the sponsors of this study played no role in designing the study; collecting, analyzing, or interpreting the data; or writing the report.

## Conflict of Interest

The authors declare that the research was conducted in the absence of any commercial or financial relationships that could be construed as a potential conflict of interest.

## Publisher’s Note

All claims expressed in this article are solely those of the authors and do not necessarily represent those of their affiliated organizations, or those of the publisher, the editors and the reviewers. Any product that may be evaluated in this article, or claim that may be made by its manufacturer, is not guaranteed or endorsed by the publisher.
