# Supplementary material for: Treatment With CD52 Antibody Protects Neurons in Experimental Autoimmune Encephalomyelitis Mice During the Recovering Phase
Source: Front Immunol. 2021 Dec 16;12:792465. doi: 10.3389/fimmu.2021.792465 (PMC8716455; doi:10.3389/fimmu.2021.792465)
Supplement: Supplementary file 1 [file DataSheet_1.pdf]

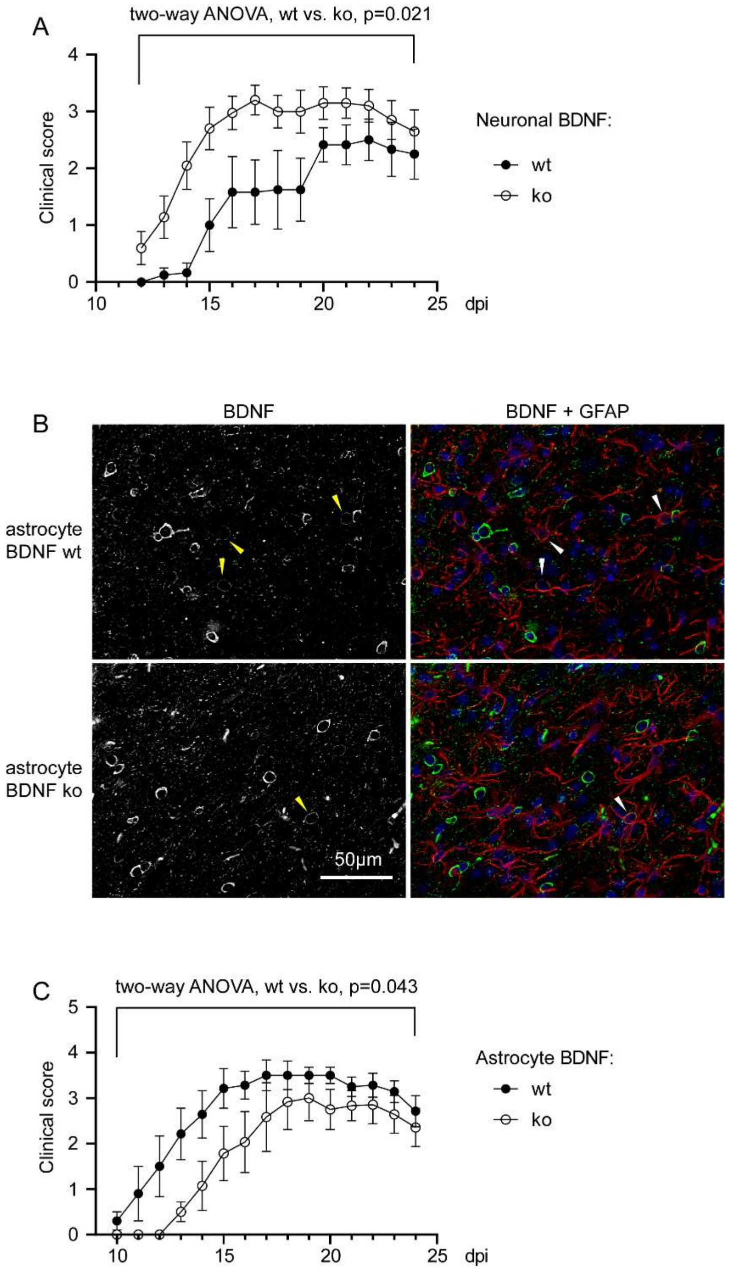

Supplementary Fig. 1, Deletion of BDNF in neurons and astrocytes differently affects EAE development in C57BL6 mice. Eight-week-old C57BL6 mice with (ko) and without (wt) BDNF deficiency specifically in neurons and astrocytes were immunized with MOG35-55 in a complete Freund's adjuvant. Clinical symptoms were monitored for more than 3 weeks. Deletion of BDNF in neurons exacerbates EAE development (A; two-way ANOVA, $F (1, 14) = 6.708$; $n \geq 6$ per group), while deletion of BDNF in astrocytes slowed down the EAE progress (C; $F (1, 12) = 5.097$; $n \geq 6$ per group), as compared with EAE mice with wildtype BDNF expression in relevant cell types. B, expression of BDNF in astrocytes was shown as colocalization (marked with arrow heads) of immunofluorescent staining of BDNF (in green) and GFAP (in red).

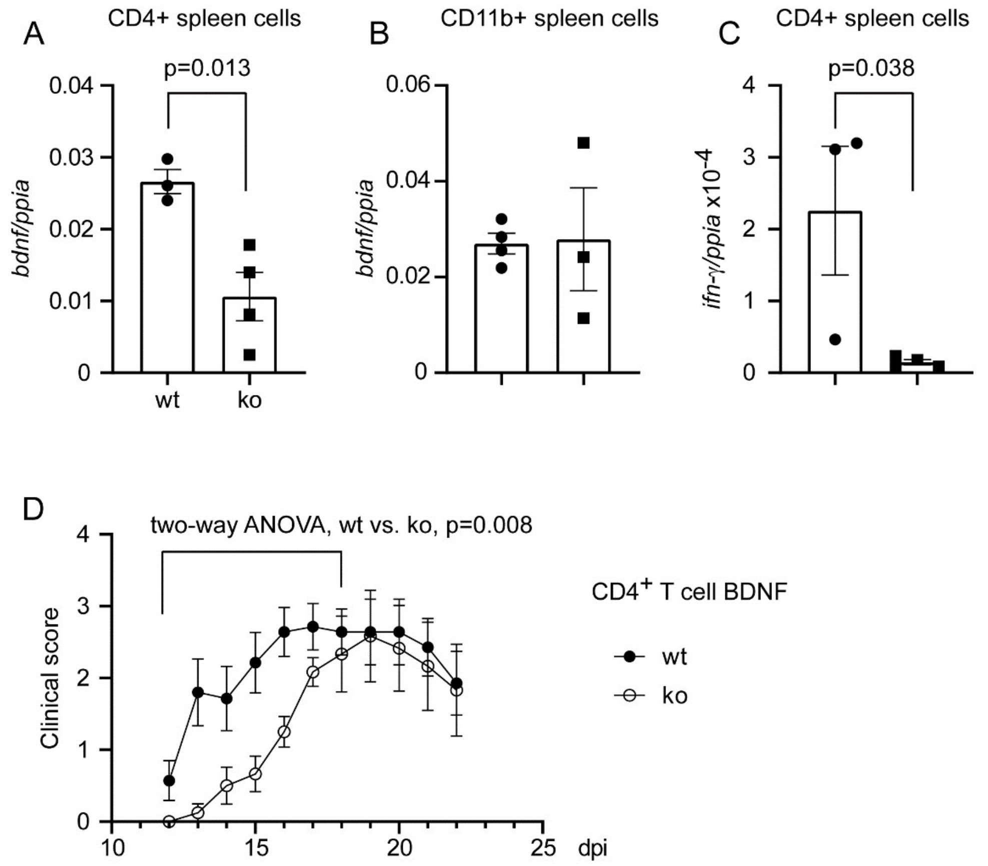

Supplementary Fig. 2, Deletion of BDNF in CD4-positive T lymphocytes attenuates EAE symptoms. Eight-week-old C57BL6 mice with (ko) and without (wt) BDNF deficiency in CD4-positive T lymphocytes were immunized with MOG35-55 in a complete Freund's adjuvant. The deletion of BDNF in lymphocytes was verified by reduced *bdnf* transcripts in CD4-positive spleen cells (A; *t* test; t (5) = 3.800; $n \geq 3$ per group). The transcription of *bdnf* gene in CD11b-positive spleen cells was not changed (B; *t* test; t (5) = 0.095; $n \geq 3$ per group). Interestingly, deletion of BDNF reduced the transcription of *inf-γ* gene in CD4-positive spleen cells compared with BDNF-wt mice (C; *t* test; t (5) = 2.809; $n \geq 3$ per group). Clinical symptoms of EAE mice were monitored for more than 3 weeks. Deletion of BDNF in CD4-positive lymphocytes significantly attenuated clinical scores of EAE (D; two-way ANOVA; F (1, 11) = 10.630; $n \geq 6$ per group).
